# Effectiveness of enzymatic hydrolysis for reducing the allergenic potential of legume by-products

**DOI:** 10.1038/s41598-022-21296-z

**Published:** 2022-10-07

**Authors:** Luisa Calcinai, Maria Giulia Bonomini, Giulia Leni, Andrea Faccini, Ilaria Puxeddu, Daiana Giannini, Fiorella Petrelli, Barbara Prandi, Stefano Sforza, Tullia Tedeschi

**Affiliations:** 1 Department of Food and Drug, University of Parma, Parco Area Delle Scienze, 27/A, 43124 Parma, Italy; 2 Centro di Servizi e Misure, University of Parma, Parco Area Delle Scienze, 23/A, Parma, Italy; 3 Immuno-Allergology Unit, Clinical and Experimental Medicine, University of Pisa, Pisa, Italy; 4 Present Address: Department of Animal Science, Food and Nutrition, Università Cattolica del Sacro Cuore, 29122 Piacenza, Italy

**Keywords:** Peptides, Proteins, Proteomics, Mass spectrometry, Biochemistry, Biological techniques, Plant sciences, Sustainability

## Abstract

The interest in agri-food residues and their valorization has grown considerably, and many of them are today considered to be valuable, under-exploited sources of different compounds and notably proteins. Despite the beneficial properties of legumes by-products, there are also some emerging risks to consider, including their potential allergenicity. In this work the immunoreactivity of chickpea, pea, and white bean by-products was assessed, and whether the production of enzymatic hydrolysates can be an effective strategy to reduce this allergenic potential. The results presented clearly indicate that the efficiency of this strategy is strongly related to the enzyme used and the food matrix. All legume by-products showed immunoreactivity towards serum of legume-allergic patients. Hydrolysates from alcalase did not show residual immunoreactivity for chickpea and green pea, whereas hydrolysates from papain still presented some immunoreactivity. However, for white beans, the presence of antinutritional factors prevented a complete hydrolysis, yielding a residual immunoreactivity even after enzymatic hydrolysis with alcalase.

## Introduction

The world population is growing steadily: the United Nations predicted that the worldwide population will increase by 1.5 billion people, reaching 9 billion by 2050, leading inevitably to a massive increase in food demand^1^. In particular, the demand for protein ingredients has considerably grown globally in the past few years, resulting in a higher interest in traditional and novel protein sources^2^. On the other side, at the same time, food industries strongly impact on the environment. In particular, the production of foods rich in animal proteins, especially meat and dairy foods, generate a much higher carbon footprint and much more consumption of natural resources (soil and water) than the production of plant-based foods^3,4^.

Thus, plant-based foods offer a great alternative to the growing world population demand and are more efficient regarding costs and resources compared to their animal-based counterparts^3,4^. Legumes represent a good source of slow-digestive starches and dietary fibers, with a small amount of fats, and these characteristics contribute to their health benefits^5^. Moreover, their high content in highly nutritional proteins makes them one of the best options to fulfill the nutritional requirements of the growing population and a valuable substitute for animal-derived protein^6,7^. For these reasons, legume production as food and feed is considerably increasing in the last few years.

Another burden on the environment imposed by the current food production systems is the generation of wastes and by-products. In recent years, still along the line of reducing the environmental burden of food production, the interest in agri-food residues and their valorization has grown considerably, and many of them are today considered to be valuable, under-exploited sources of different compounds, and notably proteins. As per the legume production chain, it generates about 400,000 tons of by-products annually^8^. They include damaged legumes, pods, leaves, stems, and hulls, which are discarded during harvesting, field processing, sorting and blanching operations^7^. Legume by-products are indeed an interesting source of functional compounds since they are rich in proteins which could be extracted and used in food formulations^4,7,9,10^. Proteins extracted from such plant discarded material may be used for formulating foods to substitute proteins of animal origin^7^.

Different methods for protein extraction from legume by-products have been proposed, either in dry conditions (milling and sieving) or wet conditions. Among these, alkaline extraction is one of the most used; however, the extreme alkaline conditions applied may denature and/or degrade the proteins and alter their functionality and digestibility. Alternatively, enzymatic-assisted extraction in water can improve the extraction yield without decreasing the nutritional value of proteins and with higher environmental sustainability in comparison with alkaline conditions^7,10^.

Despite the beneficial properties and characteristics of legumes, there are also some known and emerging risks to consider. Legumes also contain antinutritional factors which are responsible for impaired protein digestion^6^; moreover, they may contain mycotoxins and pesticide residues, thus constituting a health risk for consumers^11^. Allergenicity could represent one of the many risks associated to the increased legume consumption. Indeed, as the global consumption of pulses increases, so does the risk of eliciting allergic reactions in susceptible individuals, also to legumes not yet recognized as major allergens^12^. As a matter of fact, among the 14 allergens officially listed in Annex II of Regulation EC no 1169/2011 and thus requiring mandatory labelling if used as an ingredient, three leguminous crops are present: lupin, soybean, and peanut. However, IgE-binding (thus potentially allergenic) proteins have been identified in most legumes, namely pea, bean, lentil, and chickpea, among others^12–15^.

Allergens derived from leguminous crops mainly belong to three families: storage proteins, which include the prolamin superfamily (including non-specific lipid transfer proteins and 2S storage albumins) and the cupin superfamily (including 7S and 11S globulins), pathogenesis-related proteins (mainly constituted by homologues of the birch pollen allergen Bet v 1) and profilins^12,16^; all of them can be associated to an immediate hypersensitivity reaction with a different degree of severity^17^. The IgE-binding capacity and thus the allergenic potential of those proteins can be influenced by food processing techniques: their allergenicity may decrease, remain unchanged or even increase upon treatments such as thermal processing, enzymatic hydrolysis, and fermentation^15,18^. Among these, enzymatic treatments are among the most effective processes to reduce food allergenicity: after the hydrolysis, small peptide fragments are obtained, which are usually not recognized by the IgE-binding receptors of mast cells.

Studies on the reduction of the allergenicity of legume protein isolates can be found in the literature^19–21^. However, given the lack of a general allergenicity assessment of legume by-products materials, This work aims to test the allergenic potential of chickpea, pea, and white bean by-products, and whether the production of enzymatic hydrolysates derived from them can be an effective strategy to reduce this allergenic potential.

## Materials and methods

### Legume by-product samples

The legume samples analyzed in the present work have been supplied by Conserve Italia Soc. Coop. Agricola (San Lazzaro di Savena, Bologna, Italy) and by Conserves France (Saint Sylvestre sur Lor, France), in the frame of the EU PROLIFIC project (grant agreement No 790157). Chickpeas and white beans were rehydrated residues, while the pea residues were fresh. All the by-products were collected during specific steps of the industrial processing line, specifically sorting operations and at the exit of the blanching process, where an optical sorter divided the sample based on the color and on the dimensions. The samples were minced with a grinder and stocked at − 20 °C.

All methods were carried out in accordance with relevant guidelines.

### Enzymatic-assisted extraction of legume by-products proteins

Enzymatic-assisted extraction (EAE), according to the procedure described by Prandi et al*.*^10^, was performed in duplicate on legume by-products (chickpeas, green peas, and white beans) using commercial proteases, alcalase (AL) and papain (PA) (Sigma–Aldrich, St. Louis, MO, USA) and starting from 10 g of each legume sample. The legume matrices were finely minced with a kitchen grinder. The reaction media phosphate buffer 10 mM was brought to pH 7.5 for the hydrolysis with alcalase and to pH 6.5 for the reaction with papain. Then, 10 g of finely minced legume matrix were added to 50 ml of phosphate buffer at appropriate pH for each enzyme, together with 1% enzyme per w of legume residue. (w/w for papain which is solid, v/w for alcalase, which is a water solution). Protease activity is 300 U/ml for papain and 0.52 U/ml for alcalase respectively. The enzymatic extraction was carried out under constant stirring (with magnetic stirrer) in a water bath for 2 h, at 60 °C for the reaction carried out with alcalase, and at 65 °C for the reaction carried out with papain. The enzymes were thermally inactivated by warming up at 90 °C for 10 min. The hydrolysates were then centrifuged at 3220* g*, at 4 °C for 30 min and the protein supernatant was separated from the pellet and lyophilized.

### Molecular characterization of the proteins in legume by-products and enzymatic extracts

#### Total nitrogen by Kjeldahl method

The analysis was carried out in duplicate on 200 mg of legume by-product residues and the corresponding lyophilized enzymatic extract samples obtained after the procedure described in the previous “Enzymatic-assisted extraction of protein fraction from legume by-products” section. The protein content was determined with the Kjeldahl method, according to the AOAC International Official Methods of Analysis^22^. The final protein content of the enzymatic hydrolysates was calculated by multiplying the determined nitrogen content by 5.6^23^ as the standard nitrogen-to-protein conversion factor for legumes.

### Degree of hydrolysis (o-phtaldialdehyde analysis, OPA) (DH%) of the enzymatic extracts

The degree of hydrolysis was determined on enzymatic hydrolysates following standard procedures described in previous studies^10,24^. The degree of hydrolysis was calculated as the ratio of free nitrogen groups after hydrolysis to total nitrogen groups, derived by the amount of protein calculated by Kjeldahl method. The free nitrogen groups are calculated from their reactivity of OPA/NAC. Total nitrogen groups correspond to the total moles of nitrogen in the system and are calculated from the ratio of total grams of protein to the average molecular mass of amino acids. The molar amount of free nitrogen groups was calculated against a standard calibration curve prepared with L-isoleucine.

### SDS-PAGE polyacrylamide gel electrophoresis of legume by-products and enzymatic hydrolysates

The SDS–PAGE (Sodium Dodecyl Sulphate—Polyacrylamide Gel Electrophoresis) analysis was performed on by-product residues and enzymatic hydrolysates according to previous studies^10^. To have a fixed quantity of protein to be injected in the gel, which was 0.03 mg of protein for by-product residues and 0.05 mg of protein for hydrolysates, the quantitation was carried out with the Quant-it protein assay kit using the Qubit Fluorimeter (Invitrogen by Thermo Fisher Scientific, Rodano, MI, Italy). 10 mg of each sample were diluted in 1 ml of dithiothreitol (DTT) 5 mM (ITW Reagents Division by PanReac AppliChem, Darmstadt, Germany), urea 4 M (VWR International S.r.l., MI, Italy) and ammonium bicarbonate 100 mM (NH_4_HCO_3_) (Sigma Aldrich, St. Louis, MO, USA) buffer. The mixtures were placed on a shaker at 200 rpm for 1 h and 30 min. The samples were then centrifuged (20.000 g, at 4 °C for 10 min) and the supernatants were filtered with syringe filters (0.45 µm pore size). One microliter of the sample solution was added to 199 µl of working solution (prepared with fluorophore and protein buffer solution 1:199). Then, the by-product protein extract and the protein hydrolysates were separated on Criterion XT Bis–Tris Gel at 12% (Biorad, Hercules, CA, USA) using the running buffer XT MES 20 × (Biorad, Hercules, CA, USA). Immunodetection has been carried out with the Bio-Rad ChemiDoc MP Imaging System.

In-gel tryptic digestion analysis was performed on specific gel bands derived from SDS-PAGE analysis of white bean protein extracts and hydrolysates, following standard procedures described in previous studies^10^. For the analysis, the most intense bands in the samples were cut out from the gel. The peptides obtained from in-gel digestion were analyzed with High Resolution Mass Spectrometry (HR-MS) on the Thermo Scientific LTQ-ORBITRAP XL instrument and protein identification was carried out by using the software PEAKS (Bioinformatics Solutions Inc) and the UniProt database.

### Immunoblotting assay for allergenicity assessment

#### Human sera samples

Twelve sera samples collected from allergic patients, represented by letters A-L (Table 1), were analyzed. The samples were collected during allergy work-up in the Immuno-allergology Unit at the University of Pisa (Azienda Ospedaliero-Universitaria Pisana), Pisa, Italy. Patients’ age ranged from 20 to 78 years old. As reported in Table 1, the sera have been selected based on evidence of allergy to legumes by clinical history, *in-vivo* skin tests^25^ and/or positive specific IgE to chickpea and/or green pea and/or white bean. The *in-vivo* skin tests were performed according to the international guidelines, and the detection of specific IgE through ImmunoCAP technology (ImmunoCAP system, Phadia 250 Laboratory Systems, Uppsala, Sweden)^26^. All manufacturer’s recommendation were followed, and the calibration curves, curve controls and internal quality controls enabled validation and acceptance of the values obtained; specific IgE levels were considered positive for values > 0.10 kUA/L. The experimental protocol was approved by the Ethical Committee of the Pisa University Hospital (Approval No 19008/2021). Informed consent was obtained from all subjects.

**Table 1:** Clinical characterization of the sera of the allergic patients.

Patient code	Positive specific IgE	Positive in-vivo skin tests
A	Chickpea	None
B	Chickpea, green pea, white bean, soybean	Chickpea, green pea, leguminous mix
C	Chickpea, white bean	Chickpea, bean, soybean
D	Chickpea	None
E	Green pea, peanut	n.d
F	Green pea	n.d
G	Chickpea, green pea	Raw chickpea, cooked green pea
H	Green pea	None
I	n.d	Chickpea
J	Chickpea, white bean, soybean	Chickpea
K	Green pea, white bean, soybean	Green pea
L	Chickpea, green pea, white bean, soybean	Chickpea flour, cooked bean

Patients are coded from A to L.

n.d.: not determined.

### IgE-immunoblotting assay

Immunoblotting experiments were performed on the 12 sera of patients described above, following procedures described in previous studies^27^ with some modifications. At the end of the gel transfer run, the nitrocellulose membranes (GE Healthcare Life Sciences) were blocked with a solution of 5% bovine serum albumin (BSA) (w/v) in incubation buffer TT 1 × (Tween 20 0.3% in tris-buffered saline 10 × (TBS) solution pH 9.6) and were left under agitation for 1 h at room temperature. Non-specific primary hybridizations were performed by incubating the membranes with human sera for 1 h at room temperature. The membranes were then incubated with a solution of Goat anti-Human IgE (DyLight 680 conjugate) 1:1000 in incubation buffer TT 1 × at room temperature for 1 h, and the immunodetection was carried out with the Bio-Rad ChemiDoc MP Imaging System (Bio-Rad, Hercules, California, USA).

### Statistical analysis

Statistical analyses (one-way ANOVA, *t-*test for equal variances) were performed using Microsoft Excel version 16.30 (Redmond, Washington, USA).

## Results and discussion

### Legume by-product characterization

Chickpea, green pea, and white bean solid by-products were obtained from agro-industrial processing industries. They consisted of non-conforming skins, plant parts and pods. The chickpeas and white beans were rehydrated by the company and non-conforming products were discarded in-line during the industrial production process, whereas peas were in the fresh form.

The by-product appearance is pictured in Supplementary Fig. 1 (SF1) and their protein content is reported in Table 2.

**Table 2:** Protein content of legume by-products (% dry matter, DM) determined by Kjeldahl method.

Sample	Protein content (% DM)
Chickpea	20.3 ± 0.8
Green pea	23.7 ± 2.3
White beans	25.9 ± 1.6

All the samples showed a protein content above 20% on dry matter.

### Enzymatic-assisted extraction of protein fraction from legume by-products

EAE of proteins from legume by-products (chickpeas, peas, white beans) was investigated. The reactions were carried out using commercial proteases (papain and alcalase), already demonstrated to be very efficient in extracting proteins from legume matrices^10^. For the first time the same procedure was also applied here for white bean matrix. The reactions were performed for 2 h according to the protocol described in “Enzymatic-assisted extraction of legume by-products proteins” section and stopped by thermal inactivation of the enzymes. After that, supernatants were isolated by centrifugation and lyophilized. The efficiency of the enzymatic reactions was assessed by the determination of the protein content (Kjeldahl method) of the lyophilized protein hydrolysates, the protein extraction yield, and the determination of the degree of hydrolysis (OPA/NAC analysis). The results obtained on the protein content (% DM) (Table 3) showed that the protein content of the lyophilizates varied among the three samples and among the same sample treated with different enzymes.

**Table 3:** Protein content, protein extraction yields and degree of hydrolysis of enzyme-assisted extraction (EAE) on chickpea, green pea, and white bean.

	Sample	EAE (papain)	EAE (alcalase)
Protein content (% DM)	Chickpea	49.9 ± 0.2*	33.4 ± 0.8
	Green pea	58.2 ± 2.8*	37.6 ± 0.0
	White bean	45.1 ± 0.6*	19.4 ± 1.3
Protein extraction yield (%)^a^	Chickpea	48.8 ± 0.2*	41.4 ± 1.0
	Green pea	68.4 ± 3.3*	55.1 ± 0.0
	White bean	37.1 ± 0.5*	24.9 ± 1.1
Degree of hydrolysis (%)	Chickpea	27.8 ± 1.2	37.1 ± 0.9*
	Green pea	27.7 ± 0.4	32.5 ± 0.3
	White beans	37.2 ± 5.2	48.2 ± 8.1*

^a^Protein extraction yield has been calculated by was calculated as the ratio between the quantity of extracted proteins and the total amount of proteins in the feedstock (in %).

*statistically significant differences between the same sample treated with the two different enzymes are highlighted by the asterisk (two-tail t-test for equal variances, α = 0.05).

In all cases, consistently with previous results^10^, the samples obtained with papain showed higher protein content compared to the ones obtained with alcalase (*t*-test for equal variances, *p* < 0.05). Accordingly, papain was the enzyme extracting the highest amount of proteins, with yields ranging from 37.1% for white bean to 68.4% for green pea. These results are in agreement with the activities of the proteases, which is 1.5 units/mg for papain and 2.59 units/g for alcalase.

The DH% of protein hydrolysates was also investigated following the procedure described in “Green Pea” section. In the case of chickpea and white bean hydrolysates, the samples obtained with alcalase showed a significantly higher DH% (*t*-test for equal variances, *p* < 0.05) compared to the ones obtained with papain, while for the green pea hydrolysates the differences were statistically not significant. These results are consistent with those previously reported in the literature^10^.

It might be interesting to notice that, despite the higher DH% obtained with alcalase for all samples, the extractability of the proteins and the protein yields of the hydrolysates were higher in the case of papain, as shown in Table 3, demonstrating that protein solubilization not always strictly correlate with the degree of hydrolysis. The higher solubilization observed might be due to the higher temperature used for the enzymatic extraction in the case of papain as compared to alcalase (65 °C against 60 °C), possibly leading to a better protein solubilization even with a lower degree of hydrolysis. It can also be underlined that papain is an enzyme from vegetal source, so its activity can be improved on vegetal matrices. Anyway, it is to be underlined that in both cases the degree of hydrolysis was quite elevated (> 20%), suggestive of extremely hydrolyzed peptide mixtures composed of small oligopeptides. With these high levels of hydrolysis, peptide solubility is expected not to be affected anymore by peptide size, very small in both cases, but rather by peptide composition. Thus, the different enzyme specificity, leading to a different peptide composition in the two cases, becomes a more relevant factor for determining solubilization.

### Protein characterization by SDS-PAGE and immunological reactivity of legume by-products and the corresponding enzymatic extracts

To assess the protein composition and the immunoreactivity of the protein fractions of the legume by-products and of their hydrolysates, whole protein fractions were also analyzed by SDS-PAGE followed by immunoblotting, as described in material and methods. Protein identification was performed by in-gel tryptic digestion followed by HR-MS analyses.

#### Chickpea

**Figure 1:**
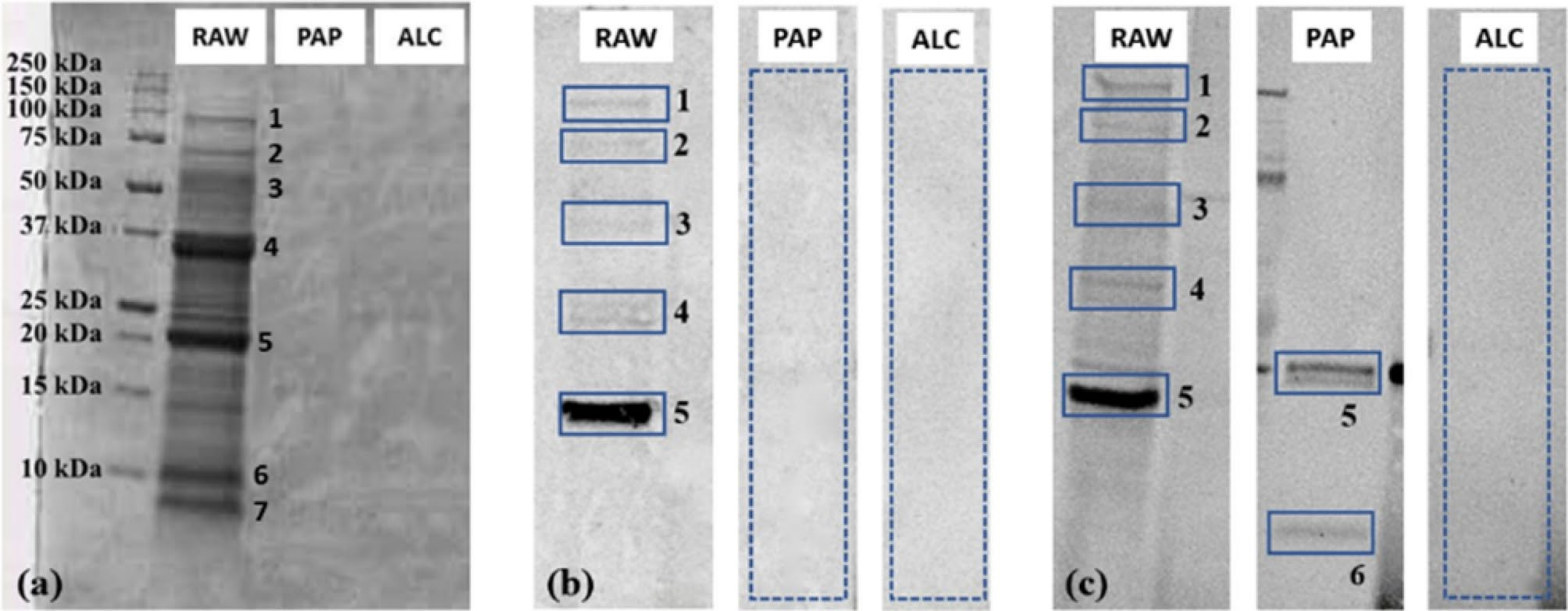
Chickpea SDS–PAGE gel (**a**) and immunoblot performed on serum E (**b**) and H (**c**) on chickpea by-product protein extract (RAW) and enzymatic hydrolysates with papain (PAP) and alcalase (ALC). Equal numbers indicate equal proteins. Original blots/gels are presented in Supplementary figures S2 for gel, S7 for blot serum E and S10 A for blot serum H.

The chickpea protein fractions obtained as described in “Enzymatic-assisted extraction of legume by-products proteins” section were analysed by SDS-PAGE following the procedure described in “Chickpea” section. Protein bands (Fig. 1a) were identified through in-gel tryptic digestion and HR-MS analysis, these were lipoxygenase (band 1), vicilin-like (bands 2, 3, 4), legumin J-like (band 5), which belongs to the 11S seed storage protein family (globulins), legumin-like (band 6) and 2S Albumin (band 7).

Chickpea hydrolysates obtained with papain (PAP) and alcalase (ALC) did not show any residual protein band, thus indicating that the enzymatic reaction produced mostly free amino acids and short peptide sequences.

Samples shown in Fig. 1a were then assessed for their allergenicity by performing immunoblotting experiments using human sera. The results of the immunoblotting assays performed on chickpea by-product extracts and enzymatic hydrolysates obtained with papain and alcalase are summarized in Supplementary Table 2 (ST2)**,** by considering positive all the samples where at least one band gave an immunological response.

The immunoblotting tests performed on chickpea samples showed IgE-binding capacity (positive reactivity) for the raw by-products, as expected. On the other side, none of the sera showed any reactivity to the enzymatic hydrolysate obtained with alcalase, whereas 5 out of 12 patient sera reacted to the enzymatic hydrolysate obtained with papain.

In Fig. 1b,c the immunoreactivity obtained with sera E and H, which are known to be immunoreactive only to green peas (Table 1) is reported as an example. Subjects A, B, G, I, J and L showed the same immunoreactivity as E, whereas subjects C, D, F and K showed the same immunoreactivity as H. As it can be seen, several protein bands immunoreacted in the raw by-product for both subjects. On the contrary, no reaction could be observed for neither subject after alcalase hydrolysis. In case of papain hydrolysis, subject H maintained some immunoreactivity, which was instead absent for subject E, also outlining a variable response according to the specific patient.

The most immunoreactive band in the by-product extract (RAW), which preserved residual immunoreactivity in the enzymatic hydrolysate obtained with papain (PAP), was identified as legumin J-like, which belongs to the 11S seed storage protein family (globulins) and it is known as the allergen Cic a 6, consistently with the literature (Bar-El Dadon et al., 2014; Wangorsch et al., 2020). Other proteins of MW ≈ 97 kDa (band 1), 70 kDa (band 2), 55 kDa (band 3) and 37 kDa (band 4) showed a less pronounced reaction. According to SDS-PAGE analysis showed in Fig. 1a and to HR-MS analysis described above, these can be identified as lipoxygenase, vicilin-like, legumin J-like and legumin-like. Band 7, whose immunoreactivity is detected in the papain hydrolysate but not in the whole by-product, was identified by HR-MS as the allergen 2S Albumin, in agreement with what was reported by Wangorsch et al.^28^. Thus, despite the overall immunoreactivity of the by-product extracts was reduced in the papain hydrolysate, with several immunoreactive bands disappearing and one highly reduced in intensity, the hydrolysis did not lead to a complete disappearance of immunoreactivity. Quite interestingly, even a new reactive protein identified as 2S Albumin (band 7) was present, which was not previously reactive in the raw material. In this case, we might hypothesize that the proteolysis unmasked new epitopes which were buried into the three-dimensional structure of the protein, therefore changing its IgE-binding capacity, and triggering a reaction after the enzymatic treatment. Thus, the above results indicate that papain may not be the most suitable enzyme for the reduction of allergenicity in this matrix, whereas alcalase seems to be able to yield a more effective hypoallergenic protein concentrate.

#### Green pea

**Figure 2:**
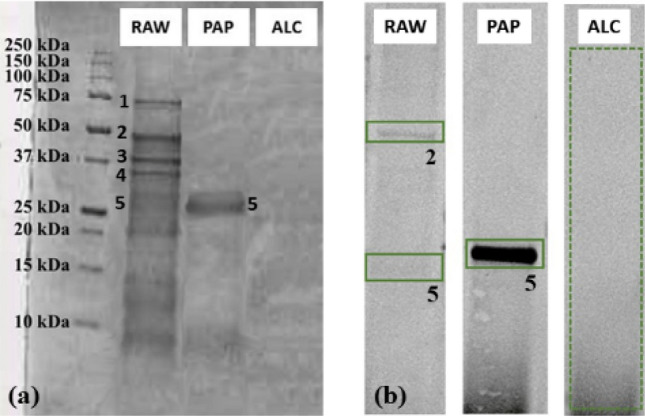
Green pea SDS–PAGE gel (**a**) and immunoblot performed on serum H (**b**) on green pea by-product protein extract (RAW) and enzymatic hydrolysates with papain (PAP) and alcalase (ALC). Equal numbers indicate equal proteins. Original blots/gels are presented in Supplementary figures S2 for gel and S10 B for blot serum H.

As for chickpea by-products (Sect. 3.2), the green pea protein fractions obtained as described in “Enzymatic-assisted extraction of protein fraction from legume by-products” section were analyzed by SDS-PAGE (Fig. 2a) and protein bands were identified through in-gel tryptic digestion and HR-MS analysis; these were convicilin (band 1), vicilin (band 2), legA class (band 3) and two vicilin 47 k subunits (band 4 and 5).

It is shown in Fig. 2a, the green pea alcalase hydrolysate (ALC) did not show any residual protein band, thus indicating that the enzymatic reaction produced mostly free amino acids and short peptide sequences, as in the case of chickpeas. A faded protein band (band 5) was detected in the papain hydrolysate (PAP); this protein was identified by HR-MS as a proteolytic fragment of pea vicilin 47 k subunit, also known as Pis s 1 allergen. This is in accordance with the literature^21^, where residual bands have been already identified in pea samples subjected to enzymatic hydrolysis.

The results of the immunoblotting assays performed on green pea by-product extract and enzymatic hydrolysates obtained with papain and alcalase are summarized in Supplementary Table 2, by considering positive all the samples where at least one band gave an immunological response.

All patients showed IgE-binding capacity (positive reactivity) for the by-product whole sample (RAW), as expected. Also, the papain hydrolysate (PAP) induced immunoreaction in all the patients’ sera, while none reacted with the one obtained with alcalase (ALC).

As all sera showed the same immunoreactivity, Fig. 2b shows the immunoblot of serum H, which reacted in the same way as all the other tested sera (A to L).

The two reactive bands in the pea residue raw sample were identified as pea vicilin (the known allergen Pis s 1) (band 2), and its αβ subunit (fragment 47 k) (band 5). The latter also gave a very strong immunoreaction in the papain hydrolysate. This is consistent with the results reported in the literature by Taylor et al.^29^, which identified vicilin as the immunodominant allergen in pea. It is interesting to observe that the band corresponding to Pis s 1 αβ subunit showed an increased intensity in the papain hydrolysate: as observed in chickpea hydrolysates, the reaction with this enzyme may have unmasked new epitopes triggering a stronger reaction in the hydrolysate.

On the other side, in the alcalase hydrolysate, no immunoreactive proteins were detected: this confirms the loss of epitopes during the reaction with this enzyme, which again seems much more effective in producing hypoallergenic hydrolysates.

#### White bean

**Figure 3:**
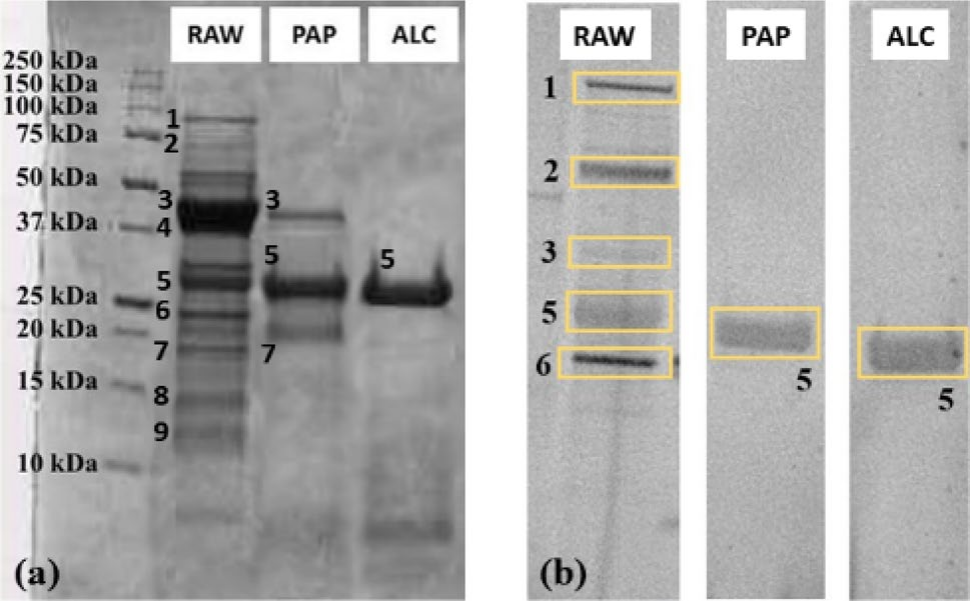
White bean SDS–PAGE gel (**a**) and immunoblot performed on serum H (**b**) on white bean by-product protein extract (RAW) and enzymatic hydrolysates with papain (PAP) and alcalase (ALC). Equal numbers indicate equal proteins. Original blots/gels are presented in Supplementary figures S2 for gel and S10 C for blot serum H.

The protein fraction of white bean by-products was identified, here for the first time, through in-gel tryptic digestion and HR-MS analysis. The most prominent bands identified for white bean (Fig. 3a) were phaseolin and its α and β types (band 3, MW 49 and 47 kDa), lectin and phytohemagglutin (leucoagglutinating and erythroagglutinating) (band 5, MW 30 kDa), and albumin-2 (band 6, MW 25 kDa).

In addition to the mass spectrometry data obtained, 9S lipoxygenase (band 1, MW 100 kDa), legumin and its acidic and basic subunits (bands 2, 4 and 7, MW 65, 37 and 20 kDa, respectively), α-amylase inhibitor (band 8, MW 18 kDa) and Bowman-Birk type protease inhibitor (band 9, MW 15 kDa) have been tentatively assigned through a comparison with previous studies based on SDS-PAGE analysis ^30^.

In this by-product, differently from what was seen in the previous legumes, residual proteins were still present in both enzymatic hydrolysates. The sample obtained with papain (PAP) showed three main bands, one of which was also prominently present in the alcalase hydrolysate (ALC). It is well known in literature that beans are particularly rich in antinutritional factors, enzyme inhibitors that could interfere with the enzymatic activity and prevent protein digestion^30^.

To identify the residual proteins in the enzymatic hydrolysates, in-gel tryptic digestion coupled with HR-MS was carried out. In accordance with the literature, band 3 was identified as the alpha polypeptide of phaseolin (MW 49 KDa), a protein which is quite resistant to enzymatic reaction^31^. Band 5 corresponded to lectin and leucoagglutinating phytohemagglutinin, antinutritional factors which are known to be resistant to enzymatic treatments mainly for their relatively compact structure^30^. Band 7 was identified, as described above, as a stable polypeptide of MW ≈ 20 kDa corresponding to a basic subunit of legumin.

The results of the immunoblotting assays performed on white bean protein extract and enzymatic hydrolysates obtained with papain and alcalase are shown in Supplementary Table 3, by considering positive all the samples where at least one band gave an immunological response.

In this case, it is quite evident that complete abolishing of immunoreactivity could not be achieved with either enzyme, as it could be supposed given the presence of proteolysis-resistant proteins. All patients showed immunoreactivity to the by-product extract and to the two hydrolysates.

Figure 3b shows the immunoblot of serum H, which behaves identically to all tested sera. Specifically, IgE-binding capacity was detected for proteins of MW ≈ 100 (band 1), 65 (band 2), 50 (band 3), 30 (band 5) and 25 kDa (band 6), identified as lipoxygenase, legumin, phaseolin, lectin and phytohemagglutinin (leucoagglutinating and erythroagglutinating), and albumin, respectively. Lectin resulted immunoreactive also after enzymatic digestion both with papain and alcalase, in agreement with the literature^30^. The results obtained agree with previous studies^32,33^ which identified the antinutritional factors lectin and phytohemagglutinins as the most allergenic proteins in bean, resistant even after thermal and enzymatic treatments. Thus, due to the inefficient hydrolysis hampered by those antinutritional factors, an efficient reduction of allergenicity in this case could not be achieved with either enzyme.

## Conclusions

According to the results shown in this work, legume by-products have the potential to trigger allergic responses in subjects presenting allergy to different legumes.

Enzymatic treatments could be employed for producing protein ingredients from legume by-products and at the same time reducing the risk of eliciting allergenic reactions in legume-sensitive individuals. Anyway, the results here presented clearly indicate that the efficacy of this strategy is strongly enzyme-related and food matrix-related.

Regarding the two enzymes used, papain seems to provide a better protein extraction yield, while alcalase is more effective in reducing the allergenicity.

For white bean samples, the presence of antinutritional factors, which also are themselves allergens, prevented a complete hydrolysis, thus also preventing the abolishing of the immunoreactivity even after enzymatic hydrolysis.

The results showed in this study confirm that protein hydrolysis can be a very effective strategy to reduce allergenicity, but also that not all the proteolytic enzymes and legume matrix yield equal results.

Further studies are needed to determine the effect of thermal processing on the immunoreactivity of the protein extracts, and on the possibility to use different enzyme combinations to achieve a better hydrolysis also in white beans, leading to hypoallergenic products.

## Supplementary Information

Supplementary Information.

## Data Availability

The datasets used and/or analysed during the current study are available from the corresponding author on reasonable request.
