# Supplementary material for: Effectiveness of enzymatic hydrolysis for reducing the allergenic potential of legume by-products
Source: Sci Rep. 2022 Oct 7;12:16902. doi: 10.1038/s41598-022-21296-z (PMC9547019; doi:10.1038/s41598-022-21296-z)
Supplement: Supplementary file 1 — Supplementary Information. [file 41598_2022_21296_MOESM1_ESM.pdf]

Supplementary material

Supplementary Table 1 (ST1) Reactivity of the sera against chickpea whole protein extract and enzymatic hydrolysates (positive reactivity at least in one protein band: +; no reactivity: -)

Serum	By-product extract	Enzymatic hydrolysate (papain)	Enzymatic hydrolysate (alcalase)
A	+	-	-
B	+	-	-
C	+	+	-
D	+	+	-
E	+	-	-
F	+	+	-
G	+	-	-
H	+	+	-
I	+	-	-
J	+	-	-
K	+	+	-
L	+	-	-

Supplementary Table 2.(ST2) Reactivity of the sera against green pea whole protein extract and enzymatic hydrolysates (positive reactivity at least in one protein band: +; no reactivity: -)

Serum	By-product extract	Enzymatic hydrolysate (papain)	Enzymatic hydrolysate (alcalase)
A	+	+	-
B	+	+	-
C	+	+	-
D	+	+	-
E	+	+	-
F	+	+	-
G	+	+	-
H	+	+	-
I	+	+	-
J	+	+	-
K	+	+	-
L	+	+	-

Supplementary Table 3. (ST3) Reactivity of the sera against cannellini bean whole protein extract and enzymatic hydrolysates (positive reactivity at least in one protein band: +; no reactivity: -)

Serum	By-product extract	Enzymatic hydrolysate (papain)	Enzymatic hydrolysate (alcalase)
A	+	+	+
B	+	+	+
C	+	+	+
D	+	+	+
E	+	+	+
F	+	+	+
G	+	+	+
H	+	+	+
I	+	+	+
J	+	+	+
K	+	+	+
L	+	+	+

Supplementary Table 4. (ST4) Protein identification after in gel triptic digestion of chickpea samples

Accession n°	Coverage (%)	Avg. mass (Da)	Description	Organism
A0A1S2XBN2	77	97,493	Lipoxygenase	*Cicer arietinum*
A0A1S2Y087	28	69,392	Vicilin-like	*Cicer arietinum*
A0A1S3E1A0	68	52,098	Vicilin-like	*Cicer arietinum*
A0A1S2XVG1	62	60,371	Legumin J-like	*Cicer arietinum*
A0A1S2XTK6	22	56,223	Legumin-like	*Cicer arietinum*
A0A1S2XDF0	10	17,099	2S albumin-like	*Cicer arietinum*

Supplementary Table 5. (ST5) Protein identification after in gel triptic digestion of pea samples

Accession n°	Coverage (%)	Avg. mass (Da)	Description	Organism
Q9M3X6	83	72,063	Convicilin	*Pisum sativum*
P13918	86	52,231	Vicilin	*Pisum sativum*
Q9T0P5	63	58,789	LegA class	*Pisum sativum*
D3VND9	69	49,515	Vicilin 47k	*Pisum sativum*
D3VNE2	56	49,664	Vicilin 47k	*Pisum sativum*

Supplementary Table 6. (ST6) Protein identification after in gel triptic digestion of white bean samples

Accession n°	Coverage (%)	Avg. mass (Da)	Description	Organism
Q43632	90	47.555	Phaseolin	*Phaseolus vulgaris*
P02853	90	47.566	Phaseolin, beta-type	*Phaseolus vulgaris*
P07219	91	49.271	Phaseolin, alpha-type	*Phaseolus vulgaris*
P05087	76	29.556	Leucoagglutinating phytohemagglutinin	*Phaseolus vulgaris*
V7C787	76	29.556	Lectin_legB domain-containing protein	*Phaseolus vulgaris*
P05088	79	29.746	Erythroagglutinating phytohemagglutinin	*Phaseolus vulgaris*
FBQXP8	60	25.449	Albumin-2	*Phaseolus vulgaris*

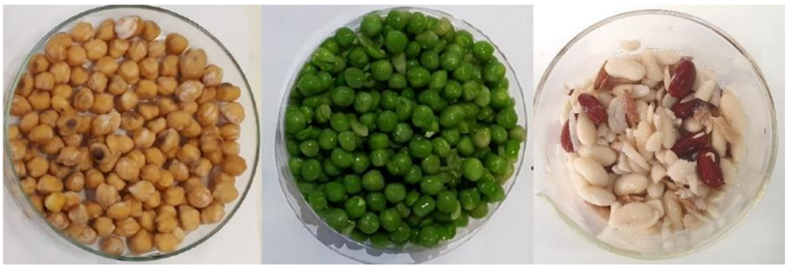

Figure S1. Chickpea, pea, and cannellini bean by-products

Additional material to the gel electrophoresis analysis

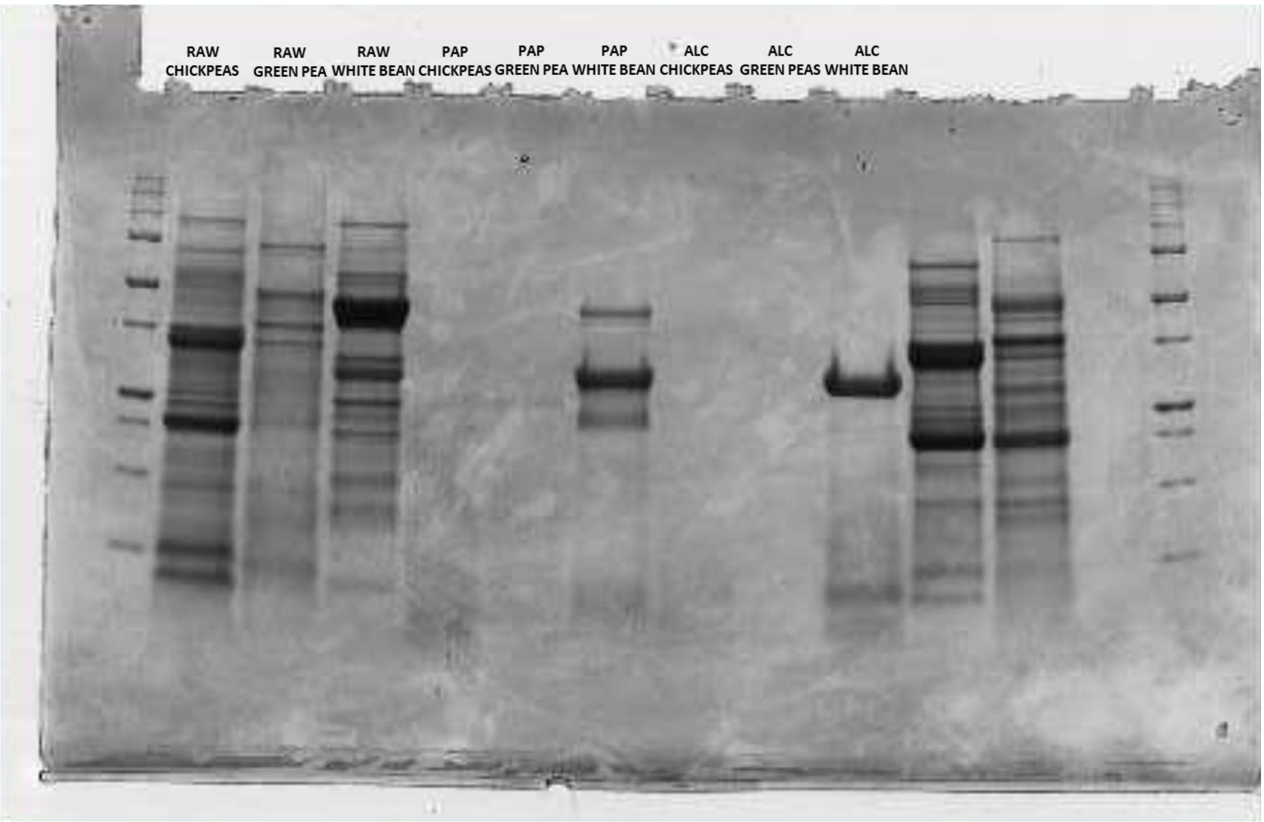

Figure S2 Full-length SDS-Page of raw chickpea, green pea and white bean and the corresponding papain (PAP) and alcalase (ALC) hydrolysates

Additional material to the blots analysis

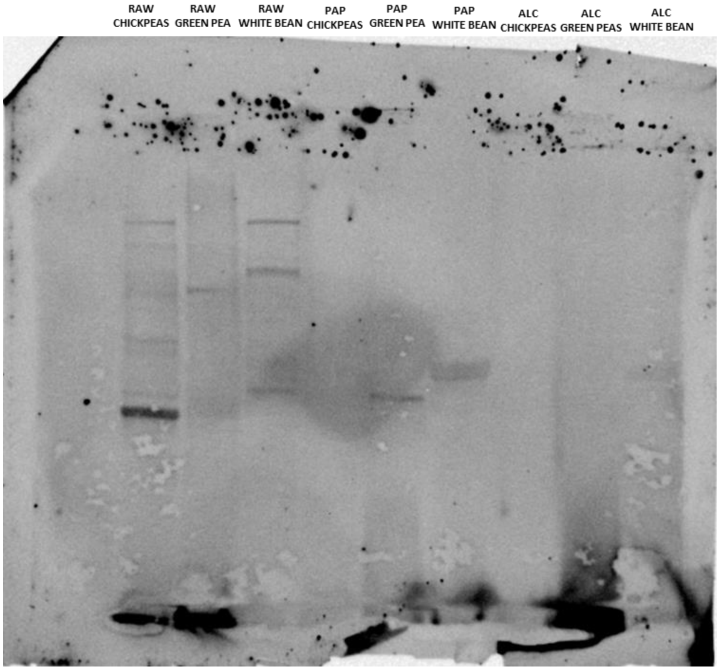

Figure S3 Full-length IgE immunoblotting of serum A

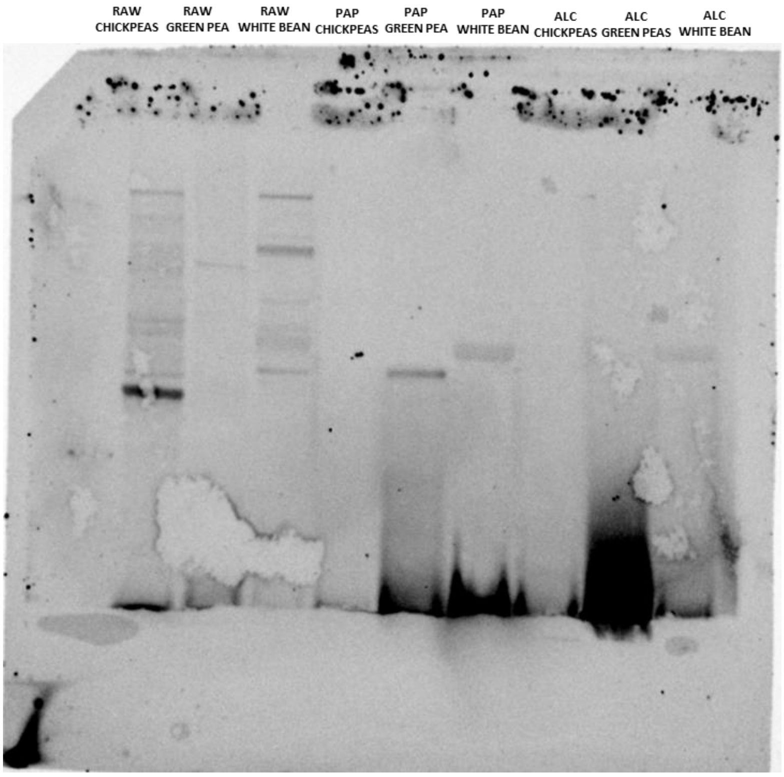

Figure S4 Full-length IgE immunoblotting of serum B

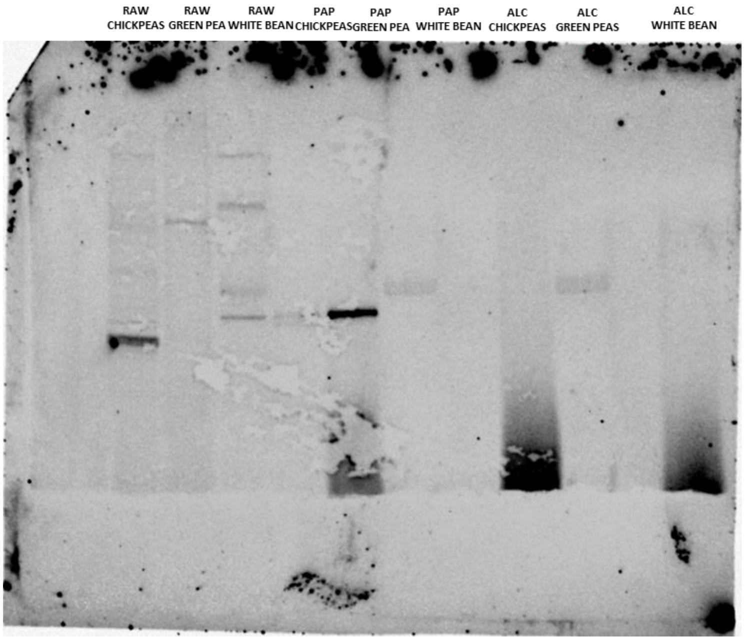

Figure S5 Full-length IgE immunoblotting of serum C

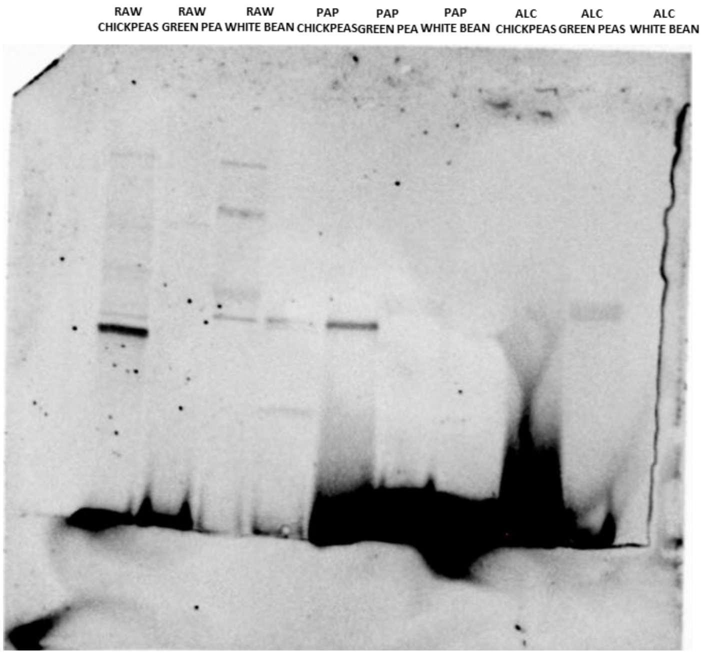

Figure S6 Full-length IgE immunoblotting of serum D

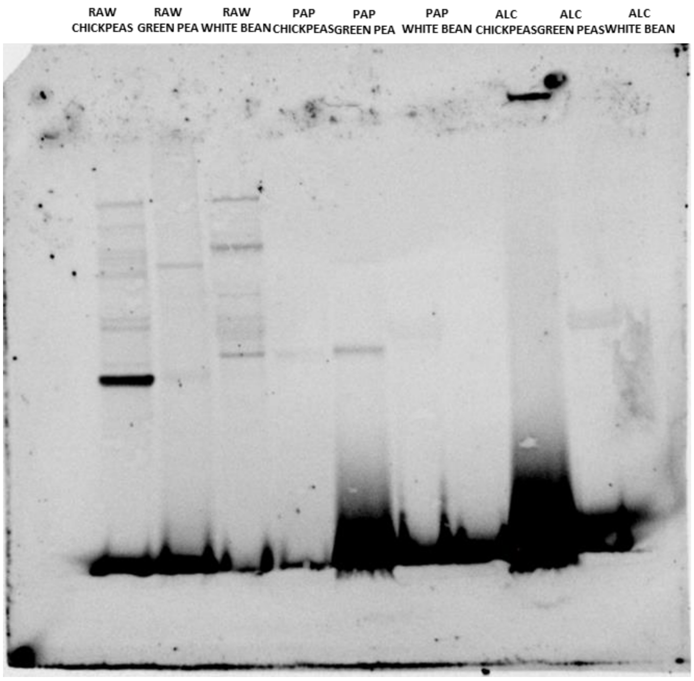

Figure S7 Full-length IgE immunoblotting of serum E

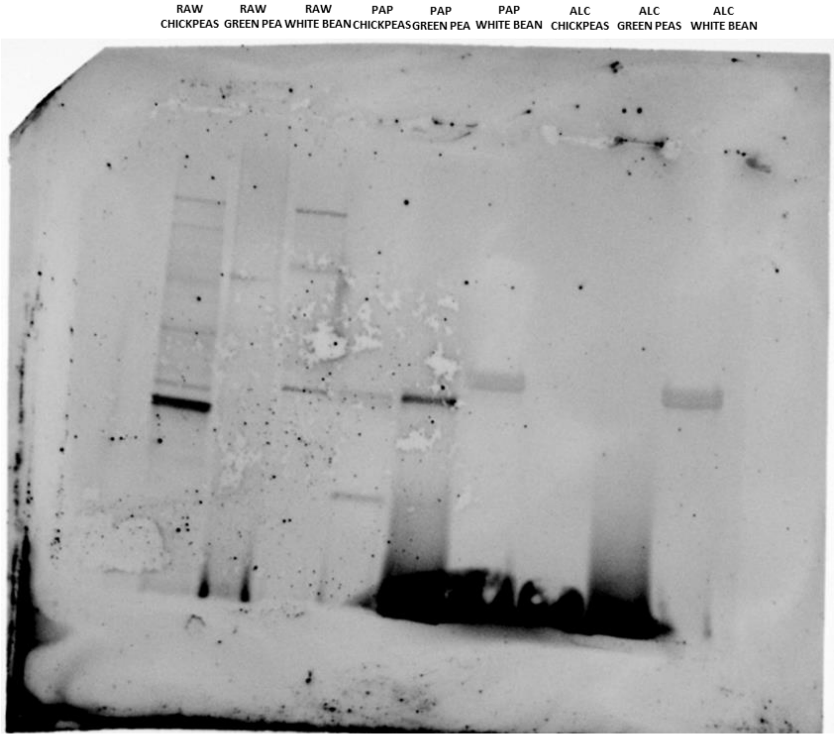

Figure S8 Full-length IgE immunoblotting of serum F

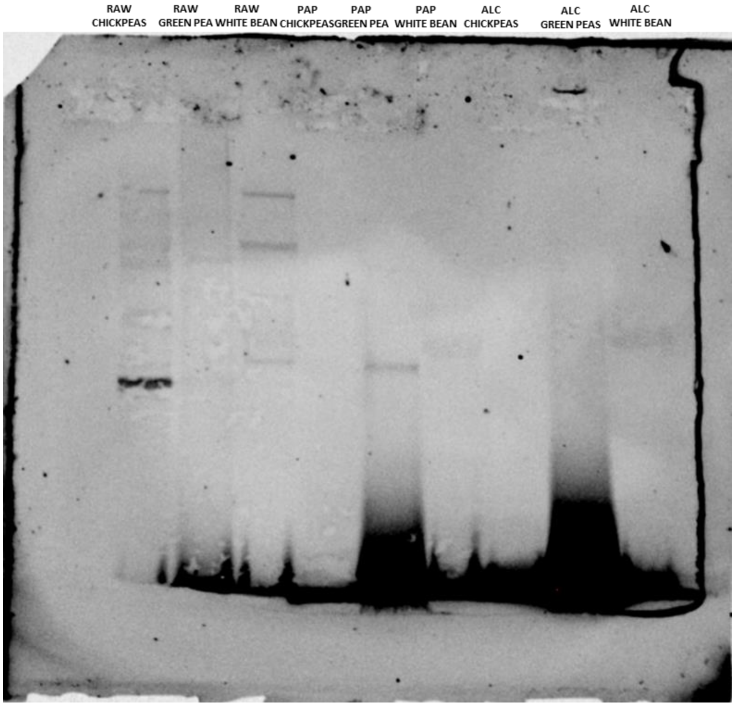

Figure S9 Full-length IgE immunoblotting of serum G

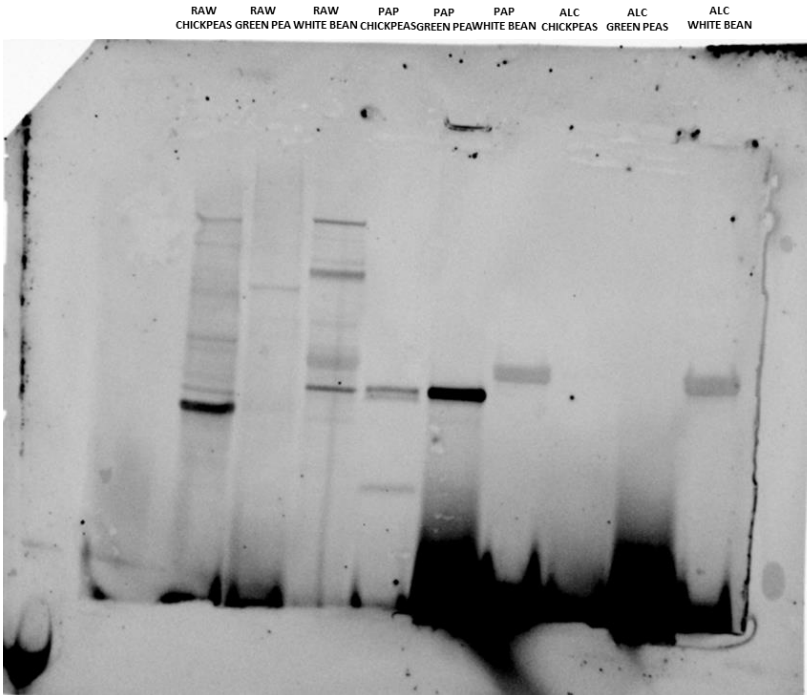

Figure S10 Full-length IgE immunoblotting of serum H

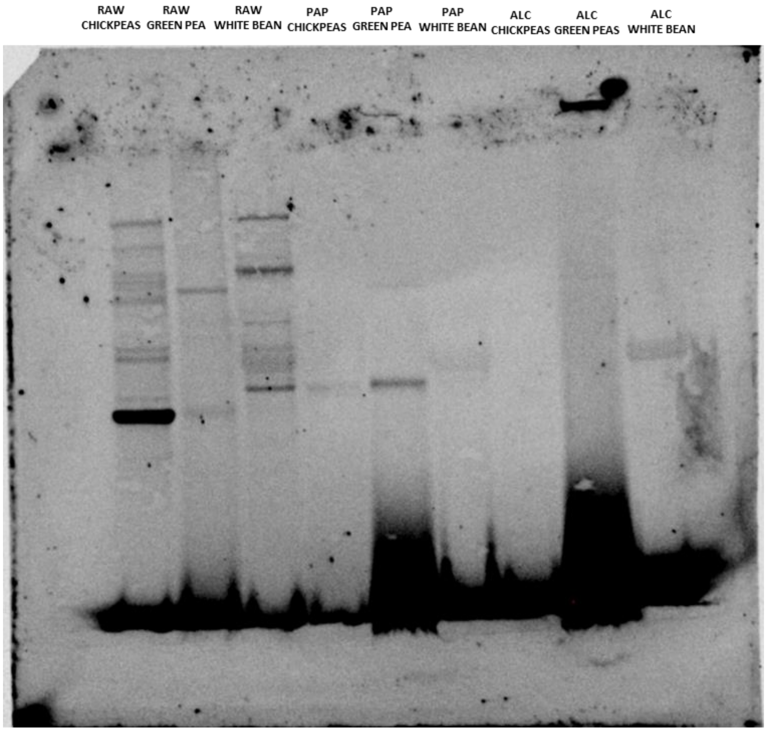

Figure S11 Full-length IgE immunoblotting of serum I

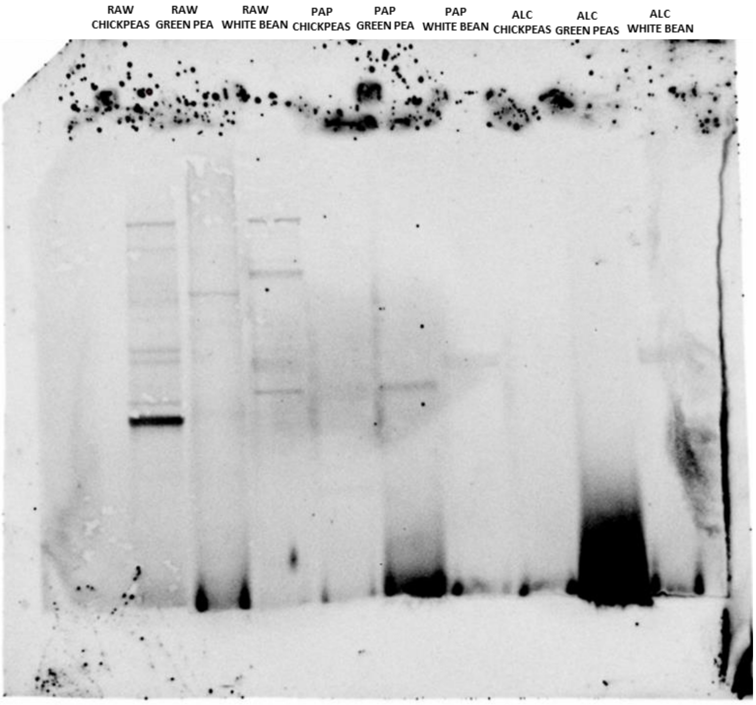

Figure S12 Full-length IgE immunoblotting of serum J

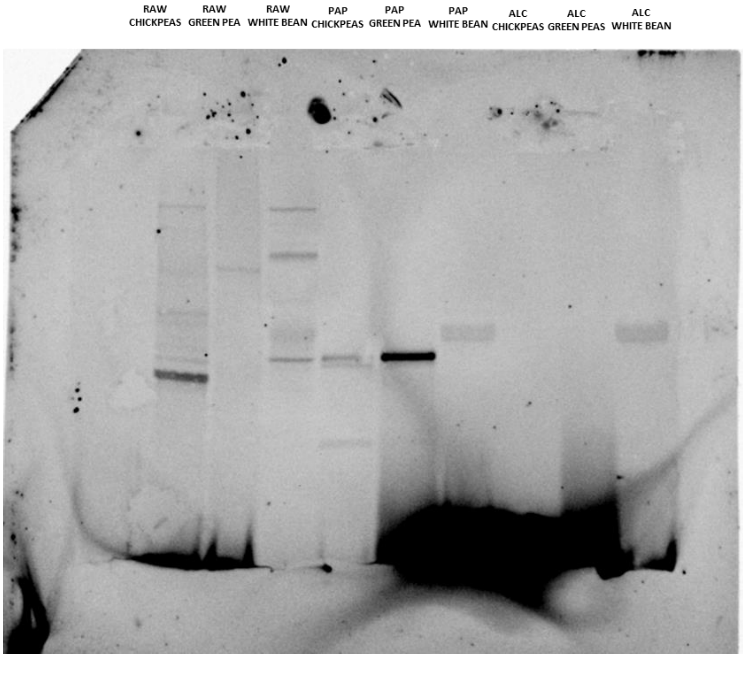

Figure S13 Full-length IgE immunoblotting of serum K

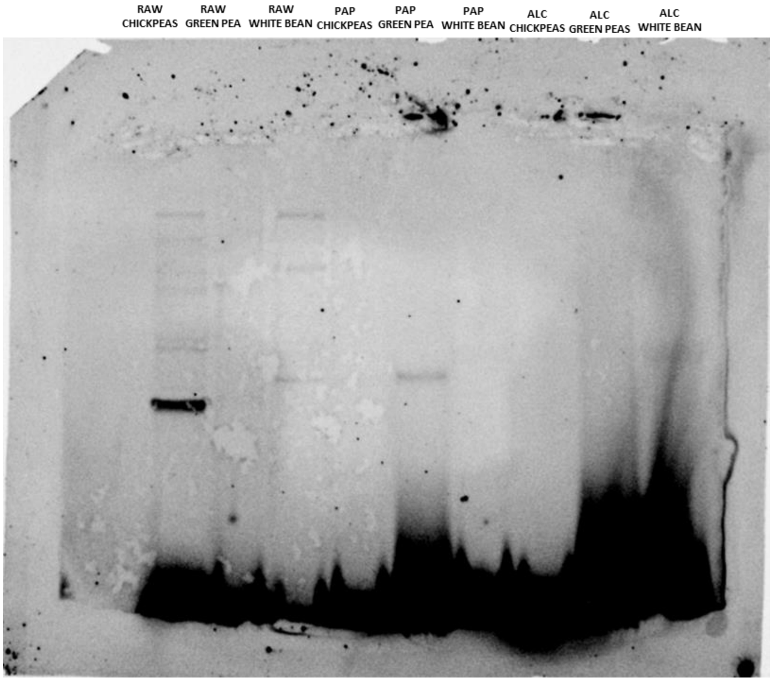

Figure S14 Full-length IgE immunoblotting of serum L
